# Towards a versatile point-of-care system combining femtosecond laser generated microfluidic channels and direct laser written microneedle arrays

**DOI:** 10.1038/s41378-019-0046-5

**Published:** 2019-02-25

**Authors:** Anika Trautmann, Gian-Luca Roth, Benedikt Nujiqi, Thomas Walther, Ralf Hellmann

**Affiliations:** 1 Applied Laser and Photonics Group, University of Applied Sciences Aschaffenburg, Wuerzburger Strasse 45, 63743 Aschaffenburg, Germany; 2 Institute of Applied Physics, Technische Universität Darmstadt, Schlossgartenstrasse 7, 64289 Darmstadt, Germany

**Keywords:** Microfluidics, Applied optics

## Abstract

Microneedle-based microfluidic systems have a great potential to become well-accepted medical devices for simple, accurate, and painless drug delivery and lab-on-a-chip diagnostics. In this work, we report on a novel hybrid approach combining femtosecond direct laser written microneedles with femtosecond laser generated microfluidic channels providing an important step towards versatile medical point-of-care systems. Hollow microneedle arrays are fabricated by a laser system designed for two-photon polymerization applications. Compression tests of two different types of truncated cone-shaped microneedle arrays prepared from OrmoComp® give information about the microneedle mechanical strength, and the results are compared to skin insertion forces. Three-dimensional microchannels are directly created inside PMMA bulk material by an ultrashort pulse laser system with vertical channels having adjustable cross-sectional areas, which allow attaching of microneedles to the microfluidic system. A comprehensive parameter study varying pulse duration and repetition rate is performed on two-photon polymerization to identify an optimal laser power range for fabricating microneedles using the same pulse duration and repetition rate as for microchannels. This addresses the advantage of a single laser system process that overcomes complex fabrication methods. A proof of concept flow test with a rhodamine B dye solution in distilled water demonstrates that the combination of microneedles and microchannels qualifies for microfluidic injection and extraction applications.

## Microfabrication: Channels and needles built by laser

Using laser light to manufacture microchannels and microneedles offers a way to greatly simplify and reduce the costs of creating these structures for drug delivery and diagnostic sampling of body fluids. Microneedle arrays and microfluidic channels are increasingly used in patches and ‘lab-on-chip’ devices that can be attached to a patient’s skin. Connecting the needles and channels allows control over the preparation, delivery, collection and analysis of fluid mixtures. Researchers in Germany, led by Ralf Hellmann at the University of Applied Sciences in Aschaffenburg, use very short pulses of laser light to fabricate suitable microchannels and microneedles from polymers. Their procedure overcomes significant complexities and limitations of current methods. The researchers demonstrate the potential of their technique using test solutions. It is now ready to be developed for real treatment and diagnosis options.

## Introduction

Point-of-care solutions based on microneedles attract interest due to their advantages in handling, patient protection, and patient care compared to conventional systems. They offer an alternative to subcutaneous medication, which, for example, is currently the preferred insulin administration method in the treatment of diabetes^1,2^. Furthermore, intradermal microneedle patches are a promising candidate to revolutionize vaccination underlining the constant demand for influenza vaccinations^2,3^ and research in cancer therapy^4,5^. Microneedle systems connected to fluidic microchannels even allow fluid extraction and meet the needs for fast and simple monitoring of physiological systems^6,7^. For all these applications, microneedle-based solutions are minimally invasive, injectable without medical experts, and minimize the risk of inflammation and infection^1,8^. Patients, especially children^5,9^, benefit from such painless^10^ and easy to handle systems improving medical care^3,11^.

Several application types of microneedles^12^ have been realized with different fabrication techniques until now. Solid microneedle^13^, coated microneedle^14^, and dissolving microneedle^15,16^ designs are distinguished to release drugs from a patch after perforating the skin or directly from the microneedle coating or dissolving microneedle into the skin. Moreover, hollow microneedles enable both drug injection^3^ and fluid extraction^6^. A cone shape^6,16,17^ or pyramid shape^7,18^ often serves as a basis for this needle type. Needle designs with offset bores prevent clogging after insertion into the skin^19^, and imitating mosquito’s fascicle is an option for optimizing the microneedle shape and reducing the insertion force^20,21^. A favorable method to fabricate these microneedles is direct laser writing via two-photon absorption^17^. In contrast to other fabrication techniques like etching methods^6,22^ or microinjection molding^23,24^, three-dimensional microneedles with arbitrary shape and high resolution can be achieved in a single-step process. This is due to the fact that photosensitive material is only modified around the focus position of the applied laser beam. OrmoComp^®^ (micro resist technology, Berlin, Germany), an organic-inorganic hybrid polymer, is an appropriate material to write microneedles with this nonlinear polymerization process showing enough hardness to penetrate the stratum corneum layer^17^ and being tested for biocompatibility^25,26^.

For accurate drug delivery and lab-on-a-chip diagnostics, microneedle arrays are combined with an additional microfluidic system. The fabrication of these systems is usually laborious and requires multiple processing steps (i.e., etching and bonding steps)^7,27–29^. Alternatively, some groups realized less complex open-channel approaches^23,30^. In this work, a femtosecond laser-based process is chosen to directly generate three-dimensional microfluidic channels inside bulk PMMA chips^31^. The idea of combining two-photon polymerization and subtractive femtosecond laser-based microfabrication is known from literature. It was used to realize microfluidic devices by ablating microgrooves in glass slabs, polymerizing microfilters inside them, and sealing the whole chip with cover glass with a single laser source^32^ or to realize micromechanical sensors by illuminating glass, etching, and two-photon polymerization^33^. To emphasize the difference to our approach, it is worth to stress that etching is a time-consuming process taking 6 h in Tičkūnas et al.^33^ and that welding is a delicate procedure^32^. Furthermore, both approaches require three main processes (ablation, polymerization, and welding or illumination, etching, and polymerization) to generate the presented devices. In notable contrast, we introduce a new approach with two main processes, microchannel creation and microneedle polymerization, in a hybrid process using only polymers and avoiding brittle^18^ and more expensive^34^ glass for the microfluidic system.

Regions of PMMA are laser modified under ambient conditions and subsequently heated to create an internal three-dimensional microfluidic system with horizontal and vertical channels. After preparation, two-photon polymerized microneedles are placed on the open vertical channels to create a combined system of microchannels and microneedles. Fluidic functionality is demonstrated by a flow test with a dye rhodamine B solution in distilled water. We highlight the joint fabrication of microneedles and microchannels with a single laser system obtained by studying direct writing for different laser parameters.

## Results

**Fig. 1:**
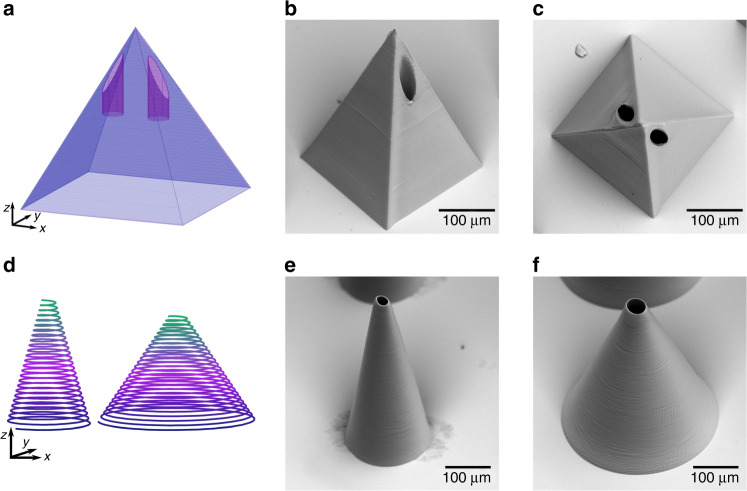
Overview of fabricated microneedle designs. CAD design **a** and SEM images showing a side view **b** and a top view **c** of a pyramid-shaped microneedle with offset bores and design **d** and SEM images showing written thin **e** and thick **f** truncated cone-shaped microneedles

Microneedles with arbitrary shape are achieved by direct laser writing via two-photon polymerization. Besides cone-shaped designs, pyramid shapes belong to the possible needle geometries. The CAD design and scanning electron microscope (SEM) images of a pyramid shape with two offset bores is shown in Fig. 1a-c. This design is more complex than common cone-shaped designs and leads to a production time of 10 min per needle. Compared to a 10 times faster fabrication of truncated cone-shaped microneedles (Fig. 1e and f) using a helix design as schematically shown in Fig. 1d, this is significantly slower.

### Mechanical compression tests

**Fig. 2:**
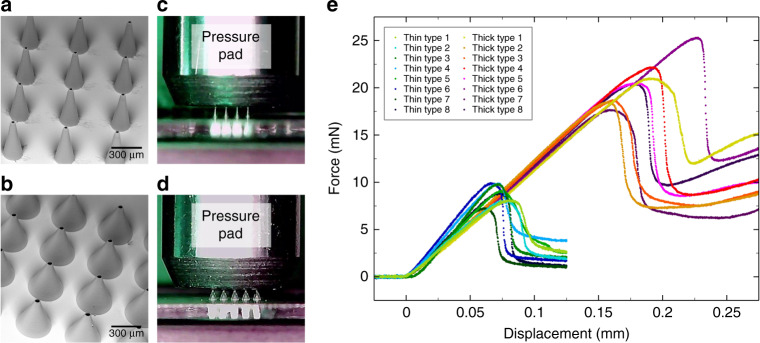
Compression tests. SEM images showing a detailed view of a thin **a** type microneedle array and a thick **b** type microneedle array, the test setup with a pressure pad lowering down onto thin **c** and thick **d** microneedle arrays, and **e** results per needle of the compression test

For two types of truncated cone-shaped microneedles on glass coverslips with laser-drilled holes, a compression test is performed. Figure 2a presents a detailed view of an array with the first type, thin microneedles, and Fig. 2b a detailed view of an array with the second type, thick microneedles, taken with a SEM. The wall angle^35^ of the microneedles is calculated by measuring the needles height, tip radius, and base radius and is 11° for thin microneedles and 24° for thick microneedles. Both needle types show a reproducible fabrication process and an exact positioning above the laser-drilled holes covered by microneedles. In each case, eight specimens are examined by a compression test. Figure 2c and d present the test setup with a pressure pad lowering down onto thin (Fig. 2c) and thick (Fig. 2d) microneedle arrays positioned above laser-drilled holes on a glass coverslip. The force and displacement of the pressure pad lowered down onto the microneedle arrays is measured and the calculated force per needle is provided in Fig. 2e. The curves start with a constant force related to the distance until the pressure pad reaches the top of the microneedles. Then, the curves increase linearly, which displays elastic deformation of the needles. After a maximum, the curves decrease again and the deviation between curves of the same type increases. The maxima of the thin needle type (7.3–9.9 mN) are smaller than the maxima of the thick needle type (17.7–25.3 mN). This points to a higher stability of the thicker microneedles. Furthermore, the maximum is a transition point from elastic to plastic deformation. The supplementary videos S1 and S2 also show that the microneedles straighten up again after compression.

**Fig. 3:**
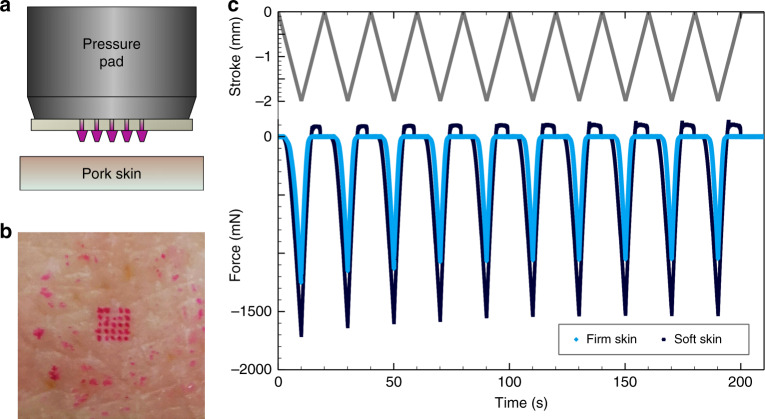
Microneedles tested against pork skin. Illustration of the test setup **a**, the impression of an array of thick microneedles onto pork skin **b**, and results of the measured force of an array of thick microneedles pressed against soft and firm pork skin, in each case performing ten iterations **c**

In a second compression test, arrays of thin and thick microneedles are fixed to a pressure pad, dyed with rhodamine B solution, and pressed against pork skin surfaces as illustrated in Fig. 3a. After the test, the pork skin is marked in the places where the microneedles interacted with the skin. The impression of a 5 × 5 array of thick microneedles is presented in Fig. 3b assuming that the middle microneedle in the first row was not sufficiently dyed. In addition, residues of pink rhodamine B solution transferred from the coverslip to the pork skin are visible surrounding the impression of the microneedle array. The microneedle specimens are tested against pork skin several times to ensure that the microneedles maintain their structural integrity after application. For penetration and permeation studies of skin, pork skin is often used as it exhibits a similar morphology as compared to human skin^36^. Figure 3c shows the results of the measured force of an array of thick microneedles pressed against soft (supplementary video S3) and firm (supplementary video S4) pork skin, in each case performing 10 iterations. The movement of the prepared pressure pad starts above the pork skin surface lowering down onto the skin with a downstroke of 2 mm. The curves show a constant force until the microneedles reach the pork skin surface after a distance of about 1 mm. Then, the curves decrease linearly due to the increasing reaction force of the skin until the turning point is reached. During the upstroke of 2 mm, the curves, in turn, increase linearly. Furthermore, there is an overshoot of the measured force observable for soft skin because the skin adheres to the coverslip. It is worth noting that the first minimum is deeper than the later ones pointing to an additional initial skin penetration force. After the compression tests, the microneedles are still intact.

### Flow test

**Fig. 4:**
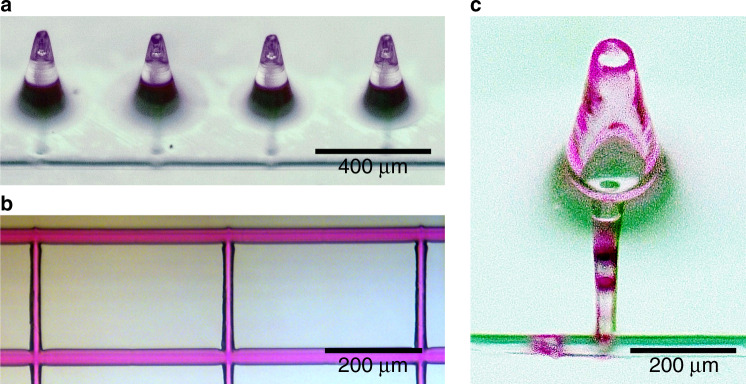
Flow test. A fluidic system combining microneedles and microchannels **a**, solution-filled microfluidic channel **b**, and microneedle with residues of pink rhodamine B solution **c**

The success of combining microneedles and microchannels using femtosecond laser-induced nonlinear processes is proved by a flow test. A fluidic system of both, needles generated by direct laser writing and channels created in a two-step process of irradiation and subsequent annealing, is shown in Fig. 4a. Four microneedles connected to a subjacent horizontal channel by vertical channels are clearly visible. The functionality of the microfluidic channels is highlighted in Fig. 4b presenting a branched network filled with a rhodamine B/distilled water solution. To ensure that liquids with different viscosity are applicable with the microneedle-based system, we successfully tested the throughput of rhodamine B/distilled water solution with a viscosity of ~1 mPa·s and of ethylene glycol with a viscosity of 20.81 mPa·s at 20 °C. Both liquids run through the openings of the vertical channels with a diameter of 30 µm, which is similar to the tip diameter of the truncated cone-shaped microneedles. Moreover, the rhodamine B/distilled water solution is injected through the opening for fluid supply into a combined system and, as expected, the solution leaks from the microneedles. A microneedle with residues of pink rhodamine B solution from the flow test is shown in Fig. 4c. This proof of principle microfluidic demonstration underlines the potential of such a microfluidic system for fluid injection and extraction.

### Parameter study

**Fig. 5:**
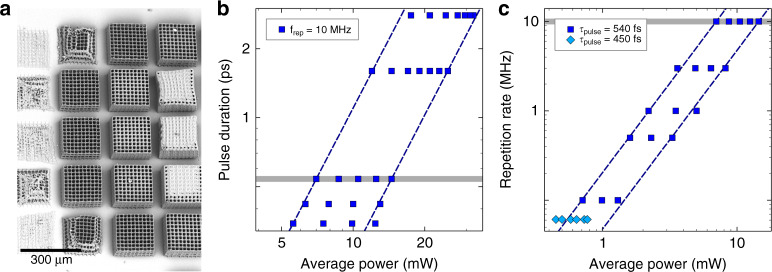
Parameter study. Three-dimensional objects with bottom-up increasing repetition rate and rightwards increasing pulse powers **a** and average laser powers for laser direct writing of microneedles with different pulse durations **b** and repetition rates **c**, whereby dashed lines show calculated power ranges based on the results for 10 MHz and 540 fs and points present successfully tested values

Microneedles as well as microfluidic channels are generated with 515 nm ultrashort pulse lasers. The processes so far only differ on repetition rate and pulse duration assuming that the laser power is adjustable. Other groups^37–39^ have shown that a variation of these two parameters is possible for two-photon polymerization. These results suggest that the repetition rate and pulse duration is adaptable for the polymerization of microneedles towards equal parameters for both laser processes. Taking account of the results of Fischer et al.^37^ for repetition rates above 100 kHz, a comparable polymerization behavior can be expected for different laser parameter settings *a* and *b* when

1 $$P_a^n\,\tau _a\,f_{rep_a} = P_b^n\,\tau _b\,f_{rep_b}$$

where *P*_*a*_ and *P*_*b*_ are the pulse powers, τ_*a*_ and τ_*b*_ are the pulse durations, $f_{rep_a}$ and $f_{rep_b}$ are the repetition rates of the laser parameter settings and *n* defines the nonlinear absorption order. Considering this equation, the nonlinear absorption order *n* of OrmoComp^®^ is determined to be two for a wavelength of 515 nm in a repetition rate range from 100 kHz to 10 MHz keeping the pulse duration constant by 540 fs and in a pulse duration range from 345 fs to 2.8 ps by a constant repetition rate of 10 MHz. For this examination, pulse powers were identified to fabricate a three-dimensional scaffold in identical quality varying the repetition rate and pulse duration using a writing speed of 1 mm/s. Figure 5a presents a part of one of the matrices used for the power determination writing scaffolds with increasing repetition rates from bottom to top and increasing power from left to right. There seems to be no influence of the repetition rate or pulse duration on the structuring quality for the investigated laser parameters. It is expected that the repetition rates and pulse durations are in a parameter window^39^, which allows an accumulation of the exposure doses^37^ of the single laser pulse in the exposure window.

Furthermore, Eq. (1) allows the calculation of a pulse power *P*_*b*_ for successful direct laser writing with a pulse duration τ_*b*_ and repetition rate $f_{rep_b}$ when an appropriate laser power *P*_*a*_ exists for direct laser writing with a pulse duration τ_*a*_ and repetition rate $f_{rep_a}$, the nonlinear absorption order *n* is known and all other parameters are equal. A laser power range to fabricate microneedles using a repetition rate of 10 MHz and a pulse duration of 540 fs is determined, and power ranges for other repetition rates and pulse durations are calculated on the basis of this result. According to Eq. (1), the calculated ranges are limited by two straight lines in a double logarithmic plot as presented with dashed lines in Fig. 5. Additionally, an overview of the selected and successfully tested values for writing microneedles is given in Fig. 5. This method for predicting an adequate laser power is extended to a repetition rate of 61 kHz and a pulse duration of 450 fs enabling the polymerization of microneedles with the same parameters and laser setup used for the microchannel generation. Figure 5c shows the power values suitable for microneedle fabrication under these conditions.

## Discussion

The combination of microneedles and microchannels as presented in this work allows the fabrication of versatile drug injection and fluid extraction systems with only a few processing steps. This is an advantage over laborious multiple processing methods^7,27–29^. It is even possible to generate both, microneedles and microchannels, using the same laser system as demonstrated in the previous section, providing the technological basis of a true hybrid process.

An important point is the penetration ability of the needles into human skin. Doraiswamy et al.^40^ performed first experiments testing OrmoComp^®^ microneedles against pork skin. After interaction with the skin, intact microneedles without fracture were observed. Moreover, Davis et al.^35^ present a comprehensive investigation of the insertion of microneedles into human skin. For microneedles with a tip radius of 30–80 µm, insertion forces from 0.08 to 3.04 N were measured. The attendant diagram^35^ indicates that the insertion force for our microneedles with a tip radius of 13 ± 2 µm (thin needles) and 21 ± 2 µm (tick needles) is expected in a range of 5 mN. This is less than the maximal force (7.3–9.9 mN for thin needles and 17.7–25.3 mN for thick needles) measured during the first compression test. Roxhed et al.^41^ designed microneedles with a sharp tip and the insertion force of a circular microneedle type was estimated to be below 10 mN. This example clearly shows that an insertion force below 10 mN is sufficient to penetrate the skin and that pyramid-shaped microneedles with a sharp tip, as presented in Fig. 1, are of interest. Altogether, laser direct written microneedles made from OrmoComp^®^ are a good choice for further microneedle-based experiments due to their stability, biocompatibility, and the ability to realize arbitrarily shaped needles.

## Materials and methods

### Microchannel generation

**Fig. 6:**
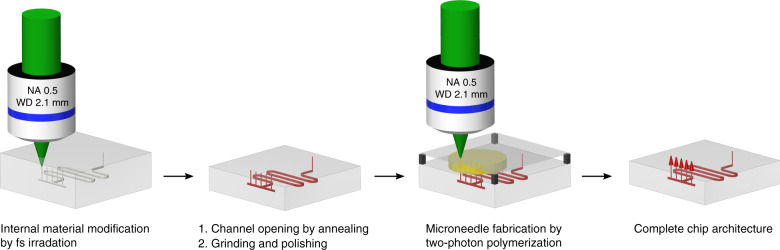
Process chart. Illustrating the combined production of internally generated microchannels by femtosecond laser irradiation and heating and microneedles created by two-photon polymerization

Microchannels are created in PMMA substrate material by a laser-induced process consisting of two process steps, outlined in Fig. 6. First, the specimens are irradiated by focused femtosecond pulses in a multi-pass process triggering a nonlinear absorption inside the focal volume. The presented process to generate hollow structures inside PMMA is based on a multi-pass scanning approach to achieve a higher microchannel quality. As also observed by Rekštytė et al.^42^, thermal accumulation, which can be attributed to a too small pulse to pulse distance, has an impact on the results of the two-photon polymerization process. We prevent this thermal accumulation by using a high scanning speed of 20 mm/s. In combination with a limited peak intensity by nonlinear beam collapsing, the multi-scanning approach is a possibility to achieve a sufficient high irradiation dose for an internal modification of PMMA. Definable 3D geometries can be created by moving the focal spot in three dimensions inside the bulk. As shown by Baum et al.^43^, femtosecond laser-irradiated PMMA exhibits a lower thermal stability as compared to pristine material. After laser processing, the specimens are placed on a hot plate between two glass substrate and are annealed at 200 °C for 30 s. Modified material is degenerated and internal microchannels are formed. It is worth noting that prior to annealing no microchannels are found, i.e., their formation is clearly linked to the annealing process. Details on this process can be found in Roth et al.^31^.

In this study, laser processing is performed using an Ytterbium-doped Potassium Gadolinium Tungstate (Yb:KGW) ultrashort pulse laser (Light Conversion, Pharos-10-600) with a wavelength of 515 nm, a pulse duration of 450 fs, and a pulse repetition rate of 61 kHz. The laser is focused by a 20x objective with an NA of 0.5 (Zeiss, EC Epiplan-Neofluar) movable along the laser beam direction by a nanopositioning z-stage (Aerotech, ANT95-50-L-Z). Samples are translated using a linear stage (Aerotech, ANT130-XY). Horizontal microchannels are generated at a writing speed of 20 mm/s and vertical microchannels at a writing speed of 10 mm/s.

### Microneedle fabrication

The microneedles are mainly written with a laser system designed for two-photon polymerization. An Ytterbium-doped femtosecond oscillator (Amplitude Systems, Mikan) having a pulse duration of 300 fs and a repetition rate of 55 MHz at 515 nm is used to realize arbitrarily shaped microneedles designed with a CAD software. For further studies, a BlueCut femtosecond fiber laser (Menlo Systems) extended by a second-harmonic generation setup was integrated. This laser system provides light pulses at a wavelength of 515 nm with adjustable pulse duration from 345 fs to 2.8 ps and variable repetition rate from 100 kHz up to 10 MHz. For direct writing, the expanded laser beam from one of the two lasers is focused by a 20x objective with an NA of 0.5 (Zeiss, EC Epiplan-Neofluar) or, alternatively, a 63x objective with an NA of 0.75 (Zeiss, LD Plan-Neofluar) into a photosensitive material layer consisting of OrmoComp^®^ (micro resist technology).

Microneedles are built up on a subjacent substrate, glass coverslip with laser-drilled holes, or prepared PMMA chip with microchannels as presented in Fig. 6 (3). A flat surface is achieved by a cover glass above the photosensitive material layer and avoids additional aberration. The samples are placed on a plate, which is movable in the plane perpendicular to the incoming beam using a translation stage (Aerotech, ANT130-XY). For real three-dimensional structures, the objective is movable along the laser beam direction by a nanopositioning z-stage (Aerotech, ANT95-50-L-Z). An integrated CCD camera qualifies to identify the holes and openings of the vertical microchannels in the substrate and to position the designed microneedles correctly onto these openings. The entire process combining direct laser written microneedle arrays and femtosecond laser generated microchannels is shown in Fig. 6.

### Mechanical compression test of microneedle arrays

Two different types of truncated cone-shaped microneedle arrays are written on glass coverslips with laser-drilled holes using a Mikan laser system and analyzed by mechanical compression tests. 4 × 4 arrays of thin microneedles (with a base radius of 100 µm, a tip radius of 15 µm, a height of 300 µm, and 500 turns) and 5 × 5 arrays of thick microneedles (with a base radius of 187.5 µm, a tip radius of 20 µm, a height of 250 µm, and 500 turns) are fabricated applying the 63x objective, a laser power of 13.5 mW, and a writing speed of 5 mm/s. This exposure of the polymer OrmoComp^®^ is followed by a post-baking for 10 min at 130 °C, developing in OrmoDev (micro resist technology) for 60 min, and hard baking for 3 h at 150 °C. One after the other, the samples are attached to the base plate of a UMT TriboLab system (Bruker), and a pressure pad with a flat surface goes down to the microneedle array at a constant speed of 0.015 mm/s. For each sample, force and displacement of the pressure pad are measured and recorded during compression.

### Microfluidic demonstration

In order to combine microneedles and microchannels, PMMA chips with an internal three-dimensional microchannel architecture are ground down to open the vertical channels. After this process step, the specimens are rinsed in an ultrasonic bath for 10 min to clean the microchannels. Before OrmoComp^®^ is applied to the samples for the two-photon polymerization process, additional openings for fluid supply, which are not covered with the photosensitive material, are temporarily closed to reduce the ingress of the polymer into the channels. A laser power of 12 mW, a repetition rate of 10 MHz, a pulse duration of 540 fs, a writing speed of 5 mm/s, and the 63x objective are appropriate to place microneedles with a base radius of 175 µm, a tip radius of 25 µm, a height of 400 µm, and 400 turns onto the channel openings. The non-polymerized material is carefully removed with distilled water to protect the PMMA chip against being damaged by OrmoDev or isopropanol. A rhodamine B/distilled water solution is injected inside the rinsed microfluidic system through the openings for fluid supply. This is observed and pictures are taken by a swingable digital microscope (Leica, DVM6).

### Variation of pulse duration and repetition rate

The extended BlueCut laser system is variable in pulse duration and repetition rate. Thus, it enables a closer examination of these parameters regarding two-photon polymerization. First, an adequate laser power range is identified to fabricate microneedles with a repetition rate of 10 MHz and a laser pulse duration of 540 fs using the 20x objective and a writing speed of 5 mm/s. The result serves as a starting point for the evaluation of other repetition rate and pulse duration values. The repetition rate is gradually reduced choosing 10, 3, 1 MHz, 500, and 100 kHz with a constant pulse duration of 540 fs. Additionally, the pulse duration is varied from 345 fs to 2.8 ps adjusting 345, 420, 540 fs, 1.6, and 2.8 ps by means of an autocorrelator (pulseCheck, APE) whereby the repetition rate is kept constant at 10 MHz. The average laser powers are measured behind the objective with an incoming beam diameter of 7.7 mm measured at 1/e^2^.

## Conclusion

In this study, a microfluidic system of direct laser written microneedles combined with internal laser generated microchannels is realized by a hybrid approach. In particular, reproducible microneedles are exactly positioned on openings of vertical microchannels, which build a branched network with horizontal channels. A successful microfluidic examination with rhodamine B solution demonstrates the applicability of such a system for fluid injection and extraction. Furthermore, the results of a compression analysis suggest that laser direct written microneedles made from OrmoComp^®^ are a promising candidate for medical microneedle applications. We identified that only a single laser system is required for both processes defining a true hybrid femtosecond laser process. This is a significant advance compared to other fabrication methods requiring several systems for multiple processing steps creating a microfluidic system with microneedles. In summary, the achieved results are a fundamental and promising basis for further research towards a versatile point-of-care system combining femtosecond laser generated microfluidic channels and direct laser written microneedle arrays.

## Supplementary information

S1

S2

S3

S4
